# Outcomes following radical prostatectomy or external beam radiation for veterans with Gleason 9 and 10 prostate cancer

**DOI:** 10.1002/cam4.4656

**Published:** 2022-03-15

**Authors:** Hann‐Hsiang Chao, Payal D. Soni, Bassam Dahman, Spiro C. Stilianoudakis, Hampton Ford, Raj Singh, Stephen J. Freedland, Drew Moghanaki, Neha Vapiwala, Michael G. Chang

**Affiliations:** ^1^ Radiation Oncology Service Hunter Holmes McGuire VA Medical Center Richmond Virginia USA; ^2^ Department of Radiation Oncology Virginia Commonwealth University Richmond Virginia USA; ^3^ Department of Health Behavior and Policy Virginia Commonwealth University Richmond Virginia USA; ^4^ Department of Biostatistics Virginia Commonwealth University Richmond Virginia USA; ^5^ Department of Surgery Cedars‐Sinai Medical Center Los Angeles California USA; ^6^ Section of Urology, Durham VA Medical Center Durham North Carolina United States; ^7^ Radiation Oncology Service VA Greater Los Angeles Healthcare System Los Angeles California USA; ^8^ Department of Radiation Oncology University of California Los Angeles Los Angeles California USA; ^9^ Department of Radiation Oncology University of Pennsylvania Philadelphia Pennsylvania USA

## Abstract

**Background:**

The optimal upfront treatment modality for patients with nonmetastatic Gleason Score 9 and 10 prostate cancer (GS 9–10 PCa) is unknown.

**Methods:**

We conducted a retrospective cohort study of patients in the Veterans Health Administration (VHA) with GS 9–10 PCa treated with radical prostatectomy (RP) or external beam radiation therapy with androgen deprivation therapy (EBRT+ADT) from 1/2000 to 12/2010. Outcomes included overall survival (OS), distant metastasis‐free survival (DMFS), and salvage/adjuvant therapy‐free survival (SAFS), as assessed by Kaplan–Meier analysis.

**Results:**

We identified 1220 veterans with GS 9–10 PCa; 335 were treated with RP, and 885 were treated with EBRT+ADT. With a median follow‐up of 9.9 years, propensity score‐matched analyses demonstrated that RP had superior 10‐year OS (70.8% [RP] vs. 61.2% [EBRT+ADT], *p* < 0.001), 10‐year DMFS rates were similar between RP (76.7%) and EBRT+ADT (81.0%), and 10‐year SAFS rates were lower for RP vs EBRT + ADT (35.2% [RP] vs. 75.2% [EBRT+ADT], *p* < 0.001). The receipt of salvage ADT was higher with upfront RP (51.9% vs. 26.1%, *p* < 0.001), despite receipt of adjuvant/salvage EBRT in 41.8% of RP patients. Among patients treated with RP, there were no differences in outcomes by race. However, higher survival rates were noted among Black patients treated with EBRT+ADT compared with White patients.

**Conclusions:**

This analysis demonstrated higher 10‐year OS rates among men treated with upfront RP versus EBRT+ADT, though missing confounders and similar DMFS rates suggest the long‐term cause‐specific OS rates may be similar. We also highlight real‐world outcomes of a diverse patient population in the VHA and improved outcomes for Black patients receiving EBRT+ADT.

## Graphical abstract

In our Veteran cohort of 1220 patients diagnosed 2000‐2010 with Gleason 9‐10 prostate cancer, patients had similar distant‐metastases free survival with either upfront RP or EBRT+ADT. Black versus White men had similar outcomes with RP and superior outcomes with EBRT+ADT. Men with Gleason score 9‐10 PC can achieve similar long‐term oncologic outcomes with either upfront RP or EBRT+ADT, but the burden and toxicities of treatment can differ depending on the treatment choice.

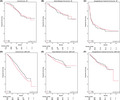

## 1 INTRODUCTION

High‐risk prostate cancer is biologically aggressive and associated with elevated rates of posttreatment relapse, metastases, and premature death compared with low‐ and intermediate‐risk disease.^1^, ^2^, ^3^ The optimal initial management strategy for high‐risk prostate cancer remains unknown but can include either upfront radical prostatectomy (RP) with or without risk‐adapted postoperative radiotherapy with or without androgen deprivation therapy (ADT), upfront external beam radiotherapy (EBRT) with long‐term ADT, or the latter plus a brachytherapy boost.^4^ Despite aggressive initial treatment, a subset of patients will ultimately develop disease recurrence, requiring salvage treatment in the form of postoperative radiation, additional ADT, or other systemic therapies. Multiple retrospective studies have demonstrated higher rates of survival with definitive RP compared with EBRT,^5^, ^6^ though these data are subject to selection bias which cannot be entirely adjusted for using statistical methods due to imbalances in baseline health that are difficult to measure. Patients undergoing RP are often younger, healthier, and have a longer overall life expectancy than patients undergoing EBRT.^7^ However, a recent large multi‐institutional report of men with Gleason score (GS) 9 and 10 prostate cancer demonstrated superior outcomes with upfront trimodality therapy with EBRT+BT with ADT relative to surgery or EBRT alone with ADT.^8^ This pooled analysis of individual patient data on 1809 patients from 12 tertiary referral centers demonstrated longer metastasis‐free survival and cancer‐specific survival among patients with GS 9–10 disease treated with trimodality therapy, raising questions about the efficacy of RP or EBRT+ADT for the management of this disease. Given the continued uncertainty regarding optimal treatment for these patients, and that there are no published or ongoing randomized trials focused specifically on GS 9–10 disease, we investigated outcomes for these patients in a retrospective cohort of men treated within a national integrated healthcare system. Additionally, as the Black population is often underrepresented in clinical trials^9^, ^10^ and comprises a significant proportion of the Veteran population, we explored whether outcomes differed by race in both the RP and EBRT+ADT cohorts within the Veterans Health Administration (VHA).

## 2 METHODS

### 2.1 Patient inclusion

As part of an institutional review board‐approved (IRB) study (study no. 1572849), which was conducted with IRB ethical approval and in accordance with recognized international standards and the principles of the Declaration of Helsinki, Veterans diagnosed with nonmetastatic prostate cancer between January 2000 and December 2010 with a biopsy GS of 9 or 10 were identified from the VHA central cancer registry. These years were chosen to ensure patients had sufficient follow‐up to assess long‐term outcomes. Patients were included if they were documented to have the clinically localized disease and received treatment with definitive intent. Patients were excluded if they received any primary treatments other than RP or EBRT (with or without brachytherapy [BT]). Common procedural terminology (CPT) codes were used to identify which patients underwent RP or EBRT, with verification of treatment receipt as necessary via manual chart abstraction. The requirement for informed consent was waived by the institutional review board.

### 2.2 Exposure

Patients were grouped into 2 cohorts based on the definitive local treatment received: RP or EBRT+ADT. Patients typically received a planned 2 years of ADT with isolated exceptions. We opted not to perform a 3 group analysis separating EBRT and EBRT+BT, due to the comparatively small subset of patients receiving BT as a component of their care and included these patients in the overall EBRT+ADT cohort.

### 2.3 Outcomes

Data regarding medications administered after the date of diagnosis were available to determine the use and timing of ADT with definitive therapy as well as in the salvage setting and confirmed by manual chart review. Salvage therapy was defined as the administration of ADT, other systemic therapies, and/or EBRT, after the development of PSA progression. Adjuvant therapy was defined as the administration of ADT and/or EBRT before a posttreatment PSA value of 0.2 mg/ml after RP or nadir +2 ng/ml after EBRT+ADT. The first date of any salvage or adjuvant systemic therapy and/or EBRT delivered was used to calculate the time of freedom from additional therapies. For EBRT+ADT patients, salvage therapy events were defined as the restart of ADT after the initial planned 2‐year duration or the start of other systemic therapies. The incidence and date of metastatic recurrence were identified by manual chart abstraction and defined as the first appearance of either radiographically or pathologically confirmed metastases, defined as nonregional nodal or distant metastases. All survival outcomes were calculated from the start date of any local treatment.

### 2.4 Statistical analysis

Descriptive statistics were used to characterize patient variables and the chi‐square test or Mann–Whitney test was used to evaluate differences in demographic, clinical, and pathological features of the patient groups. Overall survival (OS), distant metastatic‐free survival (DMFS), and salvage/adjuvant‐free survival (SAFS) were estimated using the Kaplan–Meier method and compared between groups using the log‐rank test. Propensity score adjustments were performed using multinomial logistic regression, with treatment (RP, EBRT+ADT) as the outcome, age, race, Ln (initial prostate‐specific antigen level), Gleason score, and lymph node involvement as prognostic covariates. Insufficient data were available to control for competing comorbidities between the treatment groups. A logistic regression model was used for the univariate and multivariable analyses of predictors of additional treatment, including the factors of age, race, pathologic nodal status, initial PSA, and Gleason score. Age and PSA were analyzed as continuous variables. All *p* values are two‐sided and a value of *p* < 0.05 was considered significant. All statistical analysis was performed using the R statistical package, version 4.0.3.^11^

## 3 RESULTS

### 3.1 Patient and treatment characteristics

A total of 7661 men in the VHA cancer registry were diagnosed with GS 9–10 prostate cancer between 2000 and 2010. The majority of men were excluded (*n* = 5507) for receipt of treatment outside the VA, and another subset was excluded after manual curation due to having GS 9–10 disease on the prostatectomy specimen alone, but not the pretreatment prostate biopsy (*n* = 934), leaving 1220 men for evaluation (Figure S1). The median follow‐up was 9.9 years. Patient characteristics of the study cohort are described in Table 1. Patients undergoing RP (median age 62, range 44–82) were significantly younger compared with those receiving EBRT+ADT (median age 66, range 41–88) (*p* < 0.001). Across both groups, patients were 58.9% White, 32.1% Black, and 9.0% other/not reported. There were comparatively more White patients and fewer Black patients undergoing RP vs. EBRT+ADT (64.8% vs. 56.6% and 25.4% vs. 34.7%, respectively, *p* = 0.002). Patients receiving treatment with EBRT+ADT were significantly more likely to have PSA > 20 and Gleason primary pattern 5 disease compared with surgery (31.4% vs 13.7%, *p* < 0.0001 and 12.4% and 6.6%, *p* = 0.003, respectively).

**TABLE 1:** Patient characteristics

	Total (*n* = 1220)	EBRT (*n* = 885)	Surgery (*n* = 335)	*p* value
Age (median, range)	65 (41–88)	66 (41–88)	62 (44–82)	<0.001
Race				
White	718 (58.9%)	501 (56.6%)	217 (64.8%)	0.001
Black	392 (32.1%)	307 (34.7%)	85 (25.4%)	
Other	32 (2.6%)	17 (1.9%)	15 (4.5%)	
Unknown/not reported	78 (6.4%)	60 (6.8%)	18 (5.4%)	
Clinical T‐stage				
<T2a	520 (42.6%)	487 (55.0%)	33 (9.9%)	0.002
T2b–T2c	249 (20.4%)	214 (24.2%)	35 (10.4%)	
>=T3	135 (11.1%)	125 (14.1%)	10 (3.0%)	
Unknown/not reported	316 (25.9%)	59 (6.7%)	257 (76.7%)	
Initial PSA				
<10	568 (46.6%)	373 (42.1%)	195 (58.2%)	<0.001
10–20	328 (25.9%)	234 (26.4%)	94 (28.1%)	
>20	324 (26.6%)	278 (31.4%)	46 (13.7%)	
Nodal status (any clinical or pathologic)				
Negative	1122 (92.0%)	858 (96.9%)	264 (78.8%)	<0.001
Positive	98 (8.0%)	27 (3.1%)	71 (21.2%)	
Gleason score				
4 + 5	850 (69.7%)	616 (69.6%)	234 (69.9%)	0.003
5 + 4	238 (19.5%)	159 (18.0%)	79 (23.6%)	
5 + 5	132 (10.8%)	110 (12.4%)	22 (6.6%)	

### 3.2 Disease control and survival outcomes

**FIGURE 1:**
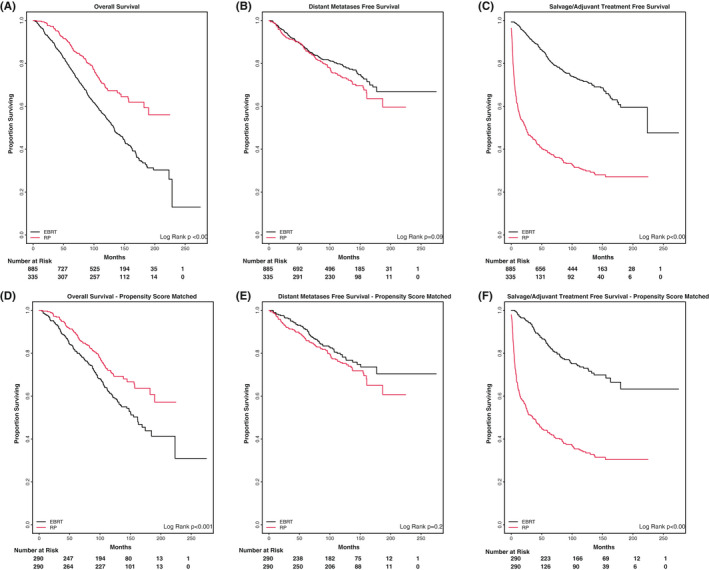
Treatment outcomes for all patients stratified by surgery and radiation, with and without propensity score matching. (A) Overall Survival, (B) distant metastases‐free survival, (C) salvage/adjuvant treatment‐free survival, (D) propensity score‐matched overall survival, (E) propensity score‐matched distant metastases‐free survival, (F) salvage/adjuvant treatment‐free survival

Ten‐year OS rates were higher among men treated with upfront RP compared with EBRT+ADT (69.0% vs. 55.1%, *p* < 0.001) (Figure 1A). Distant metastases‐free survival (DMFS) rates were not significantly different between the two groups at 10 years (74.9% vs. 81.5%, *p* = 0.09) (Figure 1B). PSA at the time of distant metastases was also not significantly different between the two groups, with a median PSA value of 22.2 for EBRT with an interquartile range of 53.3 (Q1: 6.4, Q3: 59.7) and a median PSA value of 15.7 for RP with an interquartile range of 44.2 (Q1: 5.22, Q3: 49.4) (*p* = 0.35), suggesting no bias toward earlier or later detection of metastatic disease between treatments (Table S1).

The OS compared with propensity matching was limited by insufficient information to adjust for measurable comorbidities and additional unmeasurable confounders. Nonetheless, a model was built using age, race, nodal status, initial PSA, and Gleason score as matching factors (Table S2). The analysis demonstrated higher OS at 10 years in men treated with upfront RP (70.8 vs 61.2%, *p* < 0.001) (Figure 1D). Propensity‐matched DMFS at 10 years was similar between upfront RP and EBRT+ADT (76.7% vs. 81.0%, *p* = 0.16) (Figure 1E).

**FIGURE 2:**
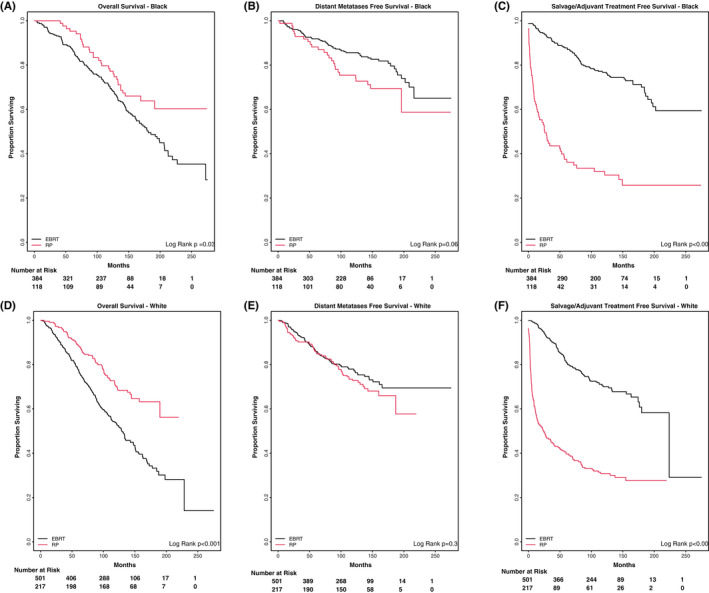
Treatment outcomes by race stratified by surgery and radiation. (A) Overall survival – Black, (B) distant metastases‐free survival – Black, (C) salvage/adjuvant treatment‐free survival – Black, (D) overall survival – White, (E) distant metastases‐free survival – White, (F) salvage/adjuvant treatment‐free survival – White

**FIGURE 3:**
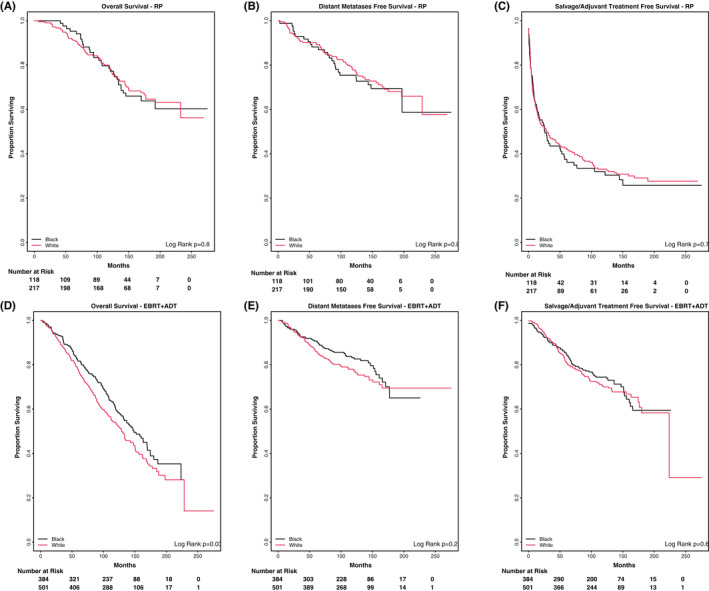
Treatment outcomes for patients stratified by race for patients undergoing radical prostatectomy (A–C), and external beam radiation (D–F). (A) Overall survival – RP, (B) distant metastases‐free survival – RP, (C) salvage/adjuvant treatment‐free survival – RP, (D) overall survival – EBRT, (E) distant metastases‐free survival – EBRT, (C) salvage/adjuvant treatment‐free survival – EBRT

Race‐stratified analyses on the overall unadjusted cohort broken out by Black and White patients mirrored the overall findings, OS significantly favoring RP and DMFS numerically, but not significantly favoring EBRT+ADT (Figure 2). We also examined survival outcomes by race within each treatment modality. Similar outcomes were seen among Black and White patients treated with upfront RP. However, Black Veterans treated with upfront EBRT+ADT were found to have a higher OS rate compared with White Veterans (10‐year OS 60.3% EBRT vs. 55.2% RP, *p* = 0.03) (Figure 3).

### 3.3 Salvage and adjuvant treatment after initial curative treatment

In patients who received initial treatment with EBRT+ADT, 231 of 885 (26.1%) subsequently received ADT for treatment failure. In RP patients, by comparison, 140 of 335 (41.8%) were treated with postoperative radiation, 174 of 335 (51.9%) were treated with subsequent ADT, 233 of 335 (69.6%) were treated with either ADT or postoperative radiation, and 81 of 335 (24.2%) required subsequent treatment with both ADT and postoperative radiation (Table 2, Figure S2). A significantly larger proportion of RP patients required treatment with any salvage/adjuvant therapy (postoperative radiation or ADT) compared with EBRT+ADT patients (233/335 [69.6%] vs. 231/885 [26.1%], *p* < 0.001) (Table 2, Figure S2). A similar proportion of RP patients ultimately required both forms of salvage/adjuvant treatment as that of EBRT patients requiring subsequent ADT (81/335 [24.2%] vs. 231/885 [26.1%], *p* = 0.5) (Table 2, Figure S2).

**TABLE 2:** Rates of additional treatment

Additional treatment needed	RP	EBRT	Fisher's exact *p* value
Any second‐line treatment	233 (69.6%)	231 (26.1%)	<0.001
Second‐line ADT	174 (51.9%)	231 (26.1%)	<0.001
Second‐line RT for RP versus second‐line ADT for EBRT	140 (41.8%)	231 (26.1%)	<0.001
Trimodality treatment for surgery versus second‐line ADT for EBRT	81 (24.2%)	231 (26.1%)	0.5

We also broke out survival outcomes by each treatment group to look at differences based on need for and type of adjuvant/salvage treatment (EBRT+ADT alone, EBRT+ADT+additional ADT, RP alone, RP+ADT, RP+RT, RT+ADT+RT) (Figure S3). Interestingly, the group receiving RP‐followed RT showed the highest OS, followed by RP alone, then RP+ADT+RT, and finally RP+ADT. For DMFS, RP alone performed the best, followed by RP+RT and RP+ADT+RT, then RP+ADT. The OS findings otherwise largely mirrored that of the overall data set, with the RP groups generally having improved OS compared with the EBRT groups. For DMFS, the EBRT and RP groups that did not need further treatment had similar DMFS, whereas the RP+RT group followed closely behind. All other groups (RP+ADT, RP+ADT+RT, and EBRT+ADT+additional ADT) had comparably poorer DMFS.

A univariate analysis was performed to identify patient and disease factors associated with a higher likelihood of requiring salvage/adjuvant treatment in each treatment group (Table 3). In RP patients, younger age (odds ratio [OR] 0.95, *p* = 0.003), nodal involvement (OR 6.19, *p* < 0.001), and higher PSA (OR 1.51, *p* = 0.03) were significantly associated with increased risk of additional salvage/adjuvant treatment. On multivariable analysis, only pathologic nodal involvement (OR 5.66, *p* < 0.001) remained significantly associated with a higher likelihood of salvage/adjuvant treatment. Similarly, in EBRT+ADT patients, younger age (OR 0.97, *p* = 0.001), nodal involvement (OR 2.73, *p* = 0.01), and higher PSA (OR 1.43, *p* < 0.001) were significantly associated with increased risk of additional salvage/adjuvant treatment. On multivariable analysis in the EBRT+ADT group, age (OR 0.97, *p* = 0.003), nodal involvement (OR 2.25, *p* = 0.04), and higher PSA (OR 1.43, *p* < 0.001) all remained significantly associated with higher likelihood of additional treatment. We also performed an analysis looking at predictive factors for each individual type of adjuvant/salvage treatment after RP (RT, ADT, and RT+ADT). For the subsequent RT group, only nodal involvement was predictive in both the univariate and multivariate analyses (OR 0.23, *p* = 0.001 and OR 0.2, *p* = 0.001, respectively). For subsequent ADT, age and nodal involvement were predictive in both the univariate (OR 1.09, *p* < 0.001 and OR 3.62, *p* < 0.001, respectively) and multivariate analyses (OR 1.31, *p* < 0.001 and OR 5.66, *p* < 0.001, respectively). For trimodality treatment, only age was predictive on the univariate and multivariate analyses (OR 0.93, *p* = 0.003 and OR 0.91, *p* = 0.001 respectively) (Table S3).

**TABLE 3:** Predictive factors for salvage/adjuvant treatment

(A) Univariate logistic regression for risk of additional treatment—EBRT			
Factor	Odds ratio	95% CI	*p* value
Age	0.97	0.95–0.99	0.001
Nodes			
No			
Yes	2.73	1.26–5.89	0.0107
Ln PSA	1.43	1.24–1.67	<0.001
Gleason score			
4 + 5			
5 + 4	1.43	0.98–2.09	0.07
5 + 5	0.98	0.61–1.58	0.95
Race			
White	1.01	0.73–1.39	0.44
Other/Unknown	0.79	0.44–1.43	0.96

(B) Univariate logistic regression for risk of additional treatment—Surgery			
Factor	Odds ratio	95% CI	*p* value
Age	0.95	0.91–0.99	0.003
Nodes			
No			
Yes	6.19	2.59–14.82	<0.001
Ln PSA	1.51	1.04–2.21	0.03
Gleason score			
4 + 5			
5 + 4	1.06	0.61–1.84	0.837
5 + 5	2.08	0.68–6.37	0.199
Race			
White	0.93	0.54–1.61	0.8
Other/Unknown	0.96	0.4–2.3	0.92

(C) Multivariate logistic regression for risk of additional treatment—EBRT			
Factor	Odds ratio	95% CI	*p* value
Age	0.97	0.95–0.99	0.003
Nodes			
No			
Yes	2.25	1.00–5.06	0.04
Ln PSA	1.43	1.23–1.67	<0.001
Gleason score			
4 + 5			
5 + 4	1.39	0.94–2.05	0.98
5 + 5	1.01	0.62–1.64	0.97
Race			
White	1.23	0.87–1.72	0.24
Other/Unknown	1.07	0.58–1.98	0.83

(D) Multivariate logistic regression for risk of additional treatment—Surgery			
Factor	Odds ratio	95% CI	*p* value
Age	0.96	0.92–1.00	0.05
Nodes			
No			
Yes	5.66	2.33–13.75	<0.001
Ln PSA	1.3	0.86–1.99	0.21
Gleason score			
4 + 5			
5 + 4	1.22	0.68–2.17	0.5
5 + 5	2.31	0.73–7.29	0.2
Race			
White	1.28	0.69–2.37	0.43
Other/Unknown	1.27	0.49–3.25	0.62

The salvage/adjuvant treatment‐free survival was significantly longer for EBRT+ADT compared with RP, with 5‐ and 10‐year salvage/adjuvant treatment‐free rates of 38.7% and 31.3% for RP patients, and 84.2% and 74.4% for EBRT+ADT patients (*p* < 0.001) (Figure 1C). Median time free from additional treatment was 23.5 months in RP patients and not reached in EBRT+ADT patients. This significant difference remained after propensity score matching with 5‐ and 10‐year salvage/adjuvant treatment‐free rates of 43.4% and 35.2% for RP patients and 85.2% and 75.2% for EBRT+ADT patients (*p* < 0.001) (Figure 1F). Median time free from additional treatment in the propensity score‐matched cohort was 35.6 months in RP patients and not reached in EBRT+ADT patients.

## 4 DISCUSSION

The utilization of RP has been increasing over the past 10–20 years as a primary treatment approach for men with GS 9 and 10 prostate cancers despite a lack of prospective data comparing it with radiotherapy options. Recent studies suggest the need for re‐evaluation of comparative outcomes between dose‐escalated radiation with ADT and RP for men with GS 9 and 10 disease.^8^, ^12^, ^13^, ^14^ To further investigate the comparative effectiveness of surgery and radiation treatments for GS 9 and 10 disease, we analyzed the VHA experience in this setting.

There are many advantages to studying prostate cancer outcomes in the VA population. The VA offers a large cohort of men with prostate cancer with a good representation of minorities as well as rural and urban populations. A shared EMR among all VA medical centers and clinics allows for robust follow‐up with detailed PSA history and medication history allowing us to capture the downstream outcomes of upfront therapy choices. It has been well established that Black men are underrepresented in prostate cancer clinical trials, and the VHA data set gives us the opportunity to examine real‐world outcomes in this population.^9^, ^10^ The equal access within the VA potentially minimizes the impact of social determinants of health that can confound clinical outcomes.

In this report, we found that RP was associated with longer OS, independent of race and after propensity score matching (Figures 1 and 2). However, a lack of measurable difference in the time to distant metastases raises questions about this finding. Multiple studies have demonstrated the challenges of retrospectively comparing OS rates between surgery and radiation, given the inherent differences in the patients who undergo these different treatments and the difficulty of adjusting for known and unknown confounders.^15^, ^16^, ^17^ There is an inherent selection bias toward RP in that only those medically fit to undergo surgery have this treatment option which is one of the many reasons that may drive differential OS outcomes. It has been proposed that metastasis‐free survival may serve as a surrogate clinical endpoint for OS in men with localized disease,^18^, ^19^, ^20^ as this outcome would track closely with prostate cancer‐related deaths. A discordance in the DMFS vs OS outcomes supports the hypothesis of uncaptured comorbidities contributing to the observed difference in OS between groups but not necessarily the rate of death due to prostate cancer. Given the upfront use of systemic therapy with ADT in the EBRT cohort that is not present with RP and its effect on PSA surveillance, we examined PSA levels at the time of metastatic failure to determine if there was ascertainment bias driving delayed detection of metastatic disease in EBRT patients leading to differential outcomes. Median PSA at the time of metastatic failure was slightly higher in EBRT+ADT vs. RP patients (22.2 vs. 15.7), though not statistically different by nonparametric means testing (*p* = 0.35).

The main goal of this study was to evaluate the overall burden of treatment between the EBRT+ADT and RP arms. Treatment failure will typically prompt additional testing and salvage therapies, both of which are associated with potential physical^21^ and financial toxicities.^22^ An understanding of the likelihood of subsequent therapies is a critical part of informed decision‐making. Our rates of salvage ADT for each treatment modality studied are comparable to those previously reported.^1^, ^12^ We do note, however, that there was no codified policy as to timing or indication for salvage treatment, but that this was based on individualized clinical decision‐making. A large multi‐institutional retrospective analysis of 487 men comparing surgery and radiation in GS 9 and 10 cancers reported salvage systemic therapy use in 30.1% and 19.7% (*p* < 0.001) of men undergoing RP and EBRT+ADT, respectively. Kishan et al. later reported on a larger cohort of over 1800 men in which they reported salvage systemic therapy use in 24.1% and 12.1% in RP and EBRT+ADT patients, respectively. Our study shows similar relationships between the two groups, with approximately double the rate of salvage ADT in RP vs. EBRT+ADT patients (51.9% and 24.2%, respectively) (Table 3). The overall higher rate of salvage systemic therapy use in our study vs. prior studies may be reflective of historical practice patterns in the study period or conversely a better ability to capture salvage ADT use due to the manner in which medications are captured by the VA EMR and longer duration of follow‐up in our study.

Another finding of our analysis was that RP patients required both postoperative EBRT and salvage ADT at a rate similar to that of EBRT patients requiring salvage ADT alone (26.1% vs. 24.2%) (Table 3). The high utilization of post‐RP radiation therapy may be viewed as a pro of this treatment approach rather than a con since many patients may opt for initial surgery due to the potential for salvage radiation. It is important, however, to note that the disease control outcomes achieved with upfront RP require additional treatment burdens and are not limited to surgery alone. Conversely, EBRT+ADT carries the recommendation for 2–3 years of ADT in all (100%) patients. The consequential adverse effects of ADT can be avoided in approximately half of the men choosing upfront RP with similar oncologic outcomes. As such, given similar DMFS rates, understanding the burden of treatment (i.e., RP often with subsequent EBRT and ADT vs. EBRT and all receiving ADT) is important for patients and providers to understand.

The analysis of the VHA data set provided the opportunity to perform race‐stratified analyses which revealed important differences between Black and White patients. Prior population‐based analyses have shown that Black men are more likely to die of prostate cancer compared with White men,^23^ though disease outcomes become more similar when controlling for access to care and standardized treatment.^24^ Our study, taking advantage of a healthcare system that provides equal access to all Veterans, supports these results with similar survival outcomes between Black and White patients in RP patients. However, in patients receiving EBRT+ADT, Black patients were found to have improved OS rates compared with White patients (Figure 3). These findings lend support to the idea that Black men may harbor differences in gene expressions and biologically distinct prostate cancer that confers increased radiosensitivity^25^, ^26^ or reflects a propensity of Black men who are medically fitter to prefer a nonsurgical approach for care.

Limitations of our study include its retrospective nature and limited ability to control for confounding factors between the two treatment groups. Notably, the clinical T‐stage was poorly captured, with different ascertainments between the EBRT+ADT and RP groups, which also limits the interpretation of our findings. There may also be clinical differences between Veterans and non‐Veterans due to combat exposures, and thus our findings may not be generalizable to the greater population. Nonetheless, this report represents one of few studies examining greater than 1200 men with GS 9–10 prostate cancer and one of the first with race‐stratified analyses. Ultimately, we cannot determine whether RP or EBRT+ADT is optimal for men with GS 9–10 disease. The higher OS seen with RP may indeed suggest that it is the better choice in men who are excellent candidates for surgery, but patients should be informed of the likelihood of needing salvage/adjuvant therapies and the overall treatment burden that may be incurred with either modality. Ultimately, unless RCT data become available, men should not be uniformly offered RP or RT without an opportunity to be evaluated for both modalities, particularly in Black men where there may be biologic differences associated with increased radiosensitivity, and in all cases should be appropriately informed of the rates, methods, and burden of both the upfront treatment and subsequent salvage regardless of which treatment option is chosen.

## CONFLICT OF INTEREST

None.

## AUTHOR CONTRIBUTIONS

Hann‐Hsiang Chao, Payal D. Soni, Drew Moghanaki, Neha Vapiwala, and Michael G. Chang: conceived the analysis. Hann‐Hsiang Chao, Payal D. Soni, and Hampton Ford: data acquisition. Hann‐Hsiang Chao, Bassam Dahman, Spiro C. Stilianoudakis, and Raj Singh: data analysis. Hann‐Hsiang Chao, Payal D. Soni, and Raj Singh: initial drafting of the work. All authors were involved in the interpretation of data. All authors were involved in editing the manuscript and had final approval of the manuscript.

## ETHICS APPROVAL STATEMENT

This work was conducted with IRB ethical approval and in accordance with recognized international standards and the principles of the Declaration of Helsinki.

## Supporting information


FigureS1

FigureS2

FigureS3

TableS1

TableS2

TableS3

## Data Availability

The data that support the findings of this study are available on request from the corresponding author. The data are not publicly available due to privacy or ethical restrictions.
