# Supplementary material for: Outcomes following radical prostatectomy or external beam radiation for veterans with Gleason 9 and 10 prostate cancer
Source: Cancer Med. 2022 Mar 15;11(15):2886–95. doi: 10.1002/cam4.4656 (PMC9359878; doi:10.1002/cam4.4656)
Supplement: Supplementary file 1 — FigureS1 [file CAM4-11-2886-s005.pdf]

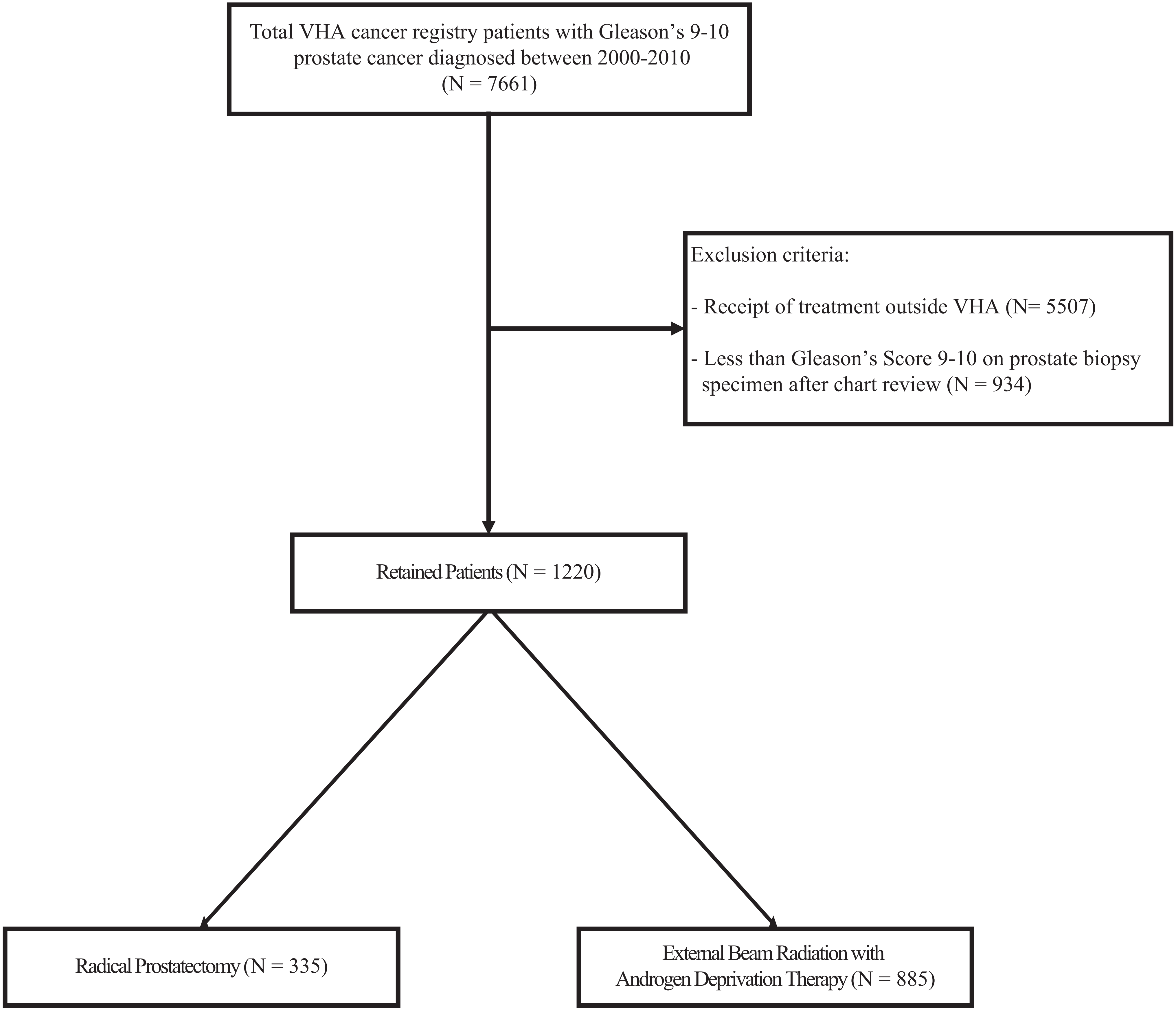
Total VHA cancer registry patients with Gleason's 9-10 prostate cancer diagnosed between 2000-2010 (N = 7661)
Exclusion criteria:
- Receipt of treatment outside VHA (N= 5507)
- Less than Gleason's Score 9-10 on prostate biopsy specimen after chart review (N = 934)
Retained Patients (N = 1220)
Radical Prostatectomy (N = 335)
External Beam Radiation with Androgen Deprivation Therapy (N = 885)
