# Supplementary figures and images for: Outcomes following radical prostatectomy or external beam radiation for veterans with Gleason 9 and 10 prostate cancer
Source: Cancer Med. 2022 Mar 15;11(15):2886–95. doi: 10.1002/cam4.4656 (PMC9359878; doi:10.1002/cam4.4656)

Supplementary Figure 2

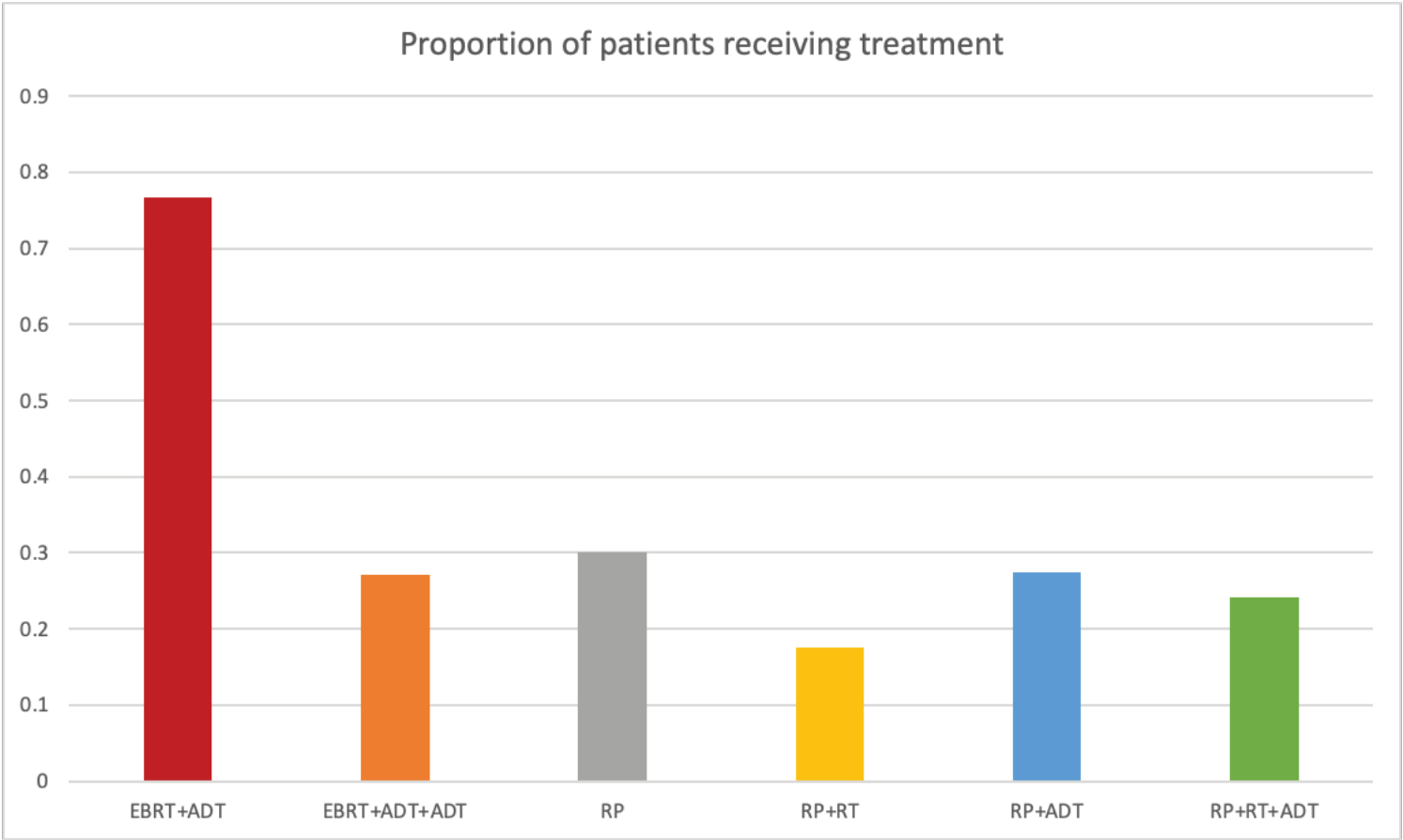

Supplement: Supplementary file 2 — FigureS2 [file CAM4-11-2886-s006.pdf]

# Supplementary Figure 3

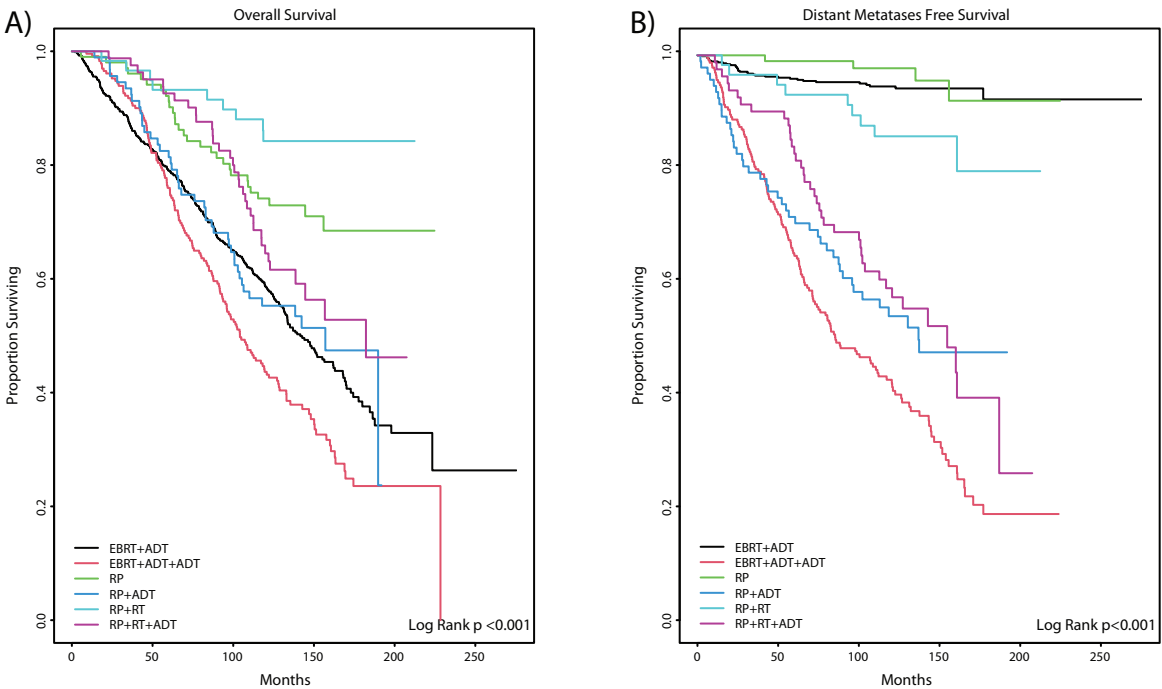

Supplement: Supplementary file 3 — FigureS3 [file CAM4-11-2886-s001.pdf]
