# Supplementary material for: Outcomes following radical prostatectomy or external beam radiation for veterans with Gleason 9 and 10 prostate cancer
Source: Cancer Med. 2022 Mar 15;11(15):2886–95. doi: 10.1002/cam4.4656 (PMC9359878; doi:10.1002/cam4.4656)
Supplement: Supplementary file 4 — TableS1 [file CAM4-11-2886-s003.docx]

**Supplementary Table 1: PSA at time of Metastatic Recurrence**

|  | EBRT | RP |
| --- | --- | --- |
| Minimum | 0.03 | 0.1 |
| Maximum | 4339 | 14508 |
| Median | 22.2 | 15.3 |
| Quartile 1 | 6.4 | 5.22 |
| Quartile 3 | 59.7 | 49.4 |
| Interquartile Range | 53.3 | 44.2 |
| Mann Whitney p-value | 0.35 | |
