# Supplementary material for: Outcomes following radical prostatectomy or external beam radiation for veterans with Gleason 9 and 10 prostate cancer
Source: Cancer Med. 2022 Mar 15;11(15):2886–95. doi: 10.1002/cam4.4656 (PMC9359878; doi:10.1002/cam4.4656)
Supplement: Supplementary file 5 — TableS2 [file CAM4-11-2886-s004.docx]

**Supplementary Table 2: Propensity Score Matched Patient Characteristics**

|  |  | Total (n=580) | | EBRT (n=290) | | Surgery (n=290) | | p-value | |  |
| --- | --- | --- | --- | --- | --- | --- | --- | --- | --- | --- |
| Age |  | 62 (44-86) | | 62 (45-86) | | 63 (44-82) | | 0.7 | |  |
| Race |  |  | |  | |  | |  | |  |
|  | White | 375 (64.7%) | | 183 (63.1%) | | 192 (66.2%) | | 0.8 | |  |
|  | Black | 145 (25.0%) | | 77 (26.6%) | | 68 (23.4%) | |  | |  |
|  | Other | 24 (4.1%) | | 11(3.8%) | | 13 (4.5%) | |  | |  |
|  | Unknown/Not Reported | 36 (6.2%) | | 19 (6.6%) | | 17 (5.9%) | |  | |  |
| Clinical T-Stage | | |  | |  | |  | |  | |
|  | <T2a | 203 (35.0%) | | 171 (59.0%) | | 32 (11.0%) | | <0.001 | |  |
|  | T2b-T2c | 89 (15.3%) | | 57 (19.7%) | | 32 (11.0%) | |  | |  |
|  | >=T3 | 53 (9.1%) | | 43 (14.8%) | | 10 (3.4%) | |  | |  |
|  | Unknown/Not Reported | 235 (40.5%) | | 19 (6.6%) | | 216(74.5%) | |  | |  |
| Initial PSA |  |  | |  | |  | |  | |  |
|  | <10 | 356 (61.4%) | | 180 (62.1%) | | 176 (60.7%) | | 0.3 | |  |
|  | 10 to 20 | 149 (25.7%) | | 68 (23.4%) | | 81 (27.9%) | |  | |  |
|  | >20 | 75 (12.9%) | | 42(14.5%) | | 33 (11.4%) | |  | |  |
| Nodal Status |  |  | |  | |  | |  | |  |
|  | No | 538 (92.8%) | | 274 (94.5%) | | 264 (91.0%) | | 0.1 | |  |
|  | Yes | 42 (7.2%) | | 16 (5.5%) | | 26 (9.0%) | |  | |  |
| Gleason Score |  |  | |  | |  | |  | |  |
|  | 4+5 | 396 (68.3%) | | 199 (68.6%) | | 197 (67.9%) | | 0.9 | |  |
|  | 5+4 | 146 (25.2%) | | 73 (25.2%) | | 73 (25.2%) | |  | |  |
|  | 5+5 | 38 (6.6%) | | 18 (6.2%) | | 20 (6.9%) | |  | |  |
