# Supplementary material for: Outcomes following radical prostatectomy or external beam radiation for veterans with Gleason 9 and 10 prostate cancer
Source: Cancer Med. 2022 Mar 15;11(15):2886–95. doi: 10.1002/cam4.4656 (PMC9359878; doi:10.1002/cam4.4656)
Supplement: Supplementary file 6 — TableS3 [file CAM4-11-2886-s002.docx]

**Supplementary Table 3: Predictive Factors For Salvage/Adjuvant Treatment after Surgery**

A)

| Univariate Logistic Regression for Risk of Additional Radiation Treatment after Surgery | | | | |
| --- | --- | --- | --- | --- |
| Factor |  | Odds Ratio | 95% CI | p-value |
| Age |  | 0.98 | 0.93 - 1.03 | 0.4 |
| Nodes |  |  |  |  |
|  | No |  |  |  |
|  | Yes | 0.23 | 0.09 - 0.56 | 0.001 |
| Ln PSA |  | 0.9 | 0.58 - 1.40 | 0.6 |
| Gleason Score |  |  |  |  |
|  | 4+5 |  |  |  |
|  | 5+4 | 0.9 | 0.44 - 1.84 | 0.8 |
|  | 5+5 | 1.12 | 0.38 - 3.32 | 0.8 |
| Race |  |  |  |  |
|  | White | 0.8 | 0.61 - 1.98 | 0.9 |
|  | Other/Unknown | 0.9 | 0.51 - 3.12 | 0.9 |

B)

| Multivariate Logistic Regression for Risk of Additional Radiation Treatment after Surgery | | | | |
| --- | --- | --- | --- | --- |
| Factor |  | Odds Ratio | 95% CI | p-value |
| Age |  | 0.98 | 0.92 - 1.03 | 0.4 |
| Nodes |  |  |  |  |
|  | No |  |  |  |
|  | Yes | 0.21 | 0.08 - 0.54 | 0.001 |
| Ln PSA |  | 1.01 | 0.60 - 1.69 | 0.98 |
| Gleason Score |  |  |  |  |
|  | 4+5 |  |  |  |
|  | 5+4 | 0.74 | 0.33 - 1.65 | 0.5 |
|  | 5+5 | 1.13 | 0.33 - 3.82 | 0.8 |
| Race |  |  |  |  |
|  | White | 0.72 | 0.43 - 1.77 | 0.9 |
|  | Other/Unknown | 0.85 | 0.66 - 3.51 | 0.9 |

C)

| Univariate Logistic Regression for Risk of Additional ADT Treatment after Surgery | | | | |
| --- | --- | --- | --- | --- |
| Factor |  | Odds Ratio | 95% CI | p-value |
| Age |  | 1.09 | 1.04 - 1.15 | <0.001 |
| Nodes |  |  |  |  |
|  | No |  |  |  |
|  | Yes | 3.62 | 1.98 - 6.64 | <0.001 |
| Ln PSA |  | 1.03 | 0.7 - 1.51 | 0.9 |
| Gleason Score |  |  |  |  |
|  | 4+5 |  |  |  |
|  | 5+4 | 1.03 | 0.55 - 1.92 | 0.9 |
|  | 5+5 | 1.23 | 0.46 - 3.28 | 0.7 |
| Race |  |  |  |  |
|  | White | 0.72 | 0.43 - 1.77 | 0.9 |
|  | Other/Unknown | 0.96 | 0.43 - 2.22 | 0.9 |

D)

| Multivariate Logistic Regression for Risk of Additional ADT Treatment after Surgery | | | | |
| --- | --- | --- | --- | --- |
| Factor |  | Odds Ratio | 95% CI | p-value |
| Age |  | 1.13 | 1.07 - 1.2 | <0.001 |
| Nodes |  |  |  |  |
|  | No |  |  |  |
|  | Yes | 5.7 | 2.96 - 11.96 | <0.001 |
| Ln PSA |  | 1.02 | 0.62 - 1.69 | 0.9 |
| Gleason Score |  |  |  |  |
|  | 4+5 |  |  |  |
|  | 5+4 | 1.21 | 0.57 - 2.57 | 0.6 |
|  | 5+5 | 1.96 | 0.58 - 6.59 | 0.3 |
| Race |  |  |  |  |
|  | White | 0.81 | 0.51 - 1.98 | 0.9 |
|  | Other/Unknown | 0.9 | 0.41 - 4.12 | 0.9 |

E)

| Univariate Logistic Regression for Risk of Trimodality Treatment with Surgery | | | | |
| --- | --- | --- | --- | --- |
| Factor |  | Odds Ratio | 95% CI | p-value |
| Age |  | 0.92 | 0.88 - 0.97 | 0.003 |
| Nodes |  |  |  |  |
|  | No |  |  |  |
|  | Yes | 0.67 | 0.35 - 1.25 | 0.2 |
| Ln PSA |  | 1.06 | 0.71 - 1.57 | 0.8 |
| Gleason Score |  |  |  |  |
|  | 4+5 |  |  |  |
|  | 5+4 | 1.06 | 0.56 - 2.1 | 0.9 |
|  | 5+5 | 0.71 | 0.24 - 2.06 | 0.5 |
| Race |  |  |  |  |
|  | White | 0.93 | 0.52 - 1.38 | 0.9 |
|  | Other/Unknown | 0.91 | 0.31 - 2.56 | 0.9 |

F)

| Multivariate Logistic Regression for Risk of Trimodality Treatment with Surgery | | | | |
| --- | --- | --- | --- | --- |
| Factor |  | Odds Ratio | 95% CI | p-value |
| Age |  | 0.91 | 0.86 - 0.96 | 0.001 |
| Nodes |  |  |  |  |
|  | No |  |  |  |
|  | Yes | 0.53 | 0.26 - 1.10 | 0.09 |
| Ln PSA |  | 0.9 | 0.57 - 1.44 | 0.7 |
| Gleason Score |  |  |  |  |
|  | 4+5 |  |  |  |
|  | 5+4 | 1.08 | 0.52 - 2.24 | 0.8 |
|  | 5+5 | 0.44 | 0.13 - 1.52 | 0.2 |
| Race |  |  |  |  |
|  | White | 0.95 | 0.51 - 1.45 | 0.9 |
|  | Other/Unknown | 0.96 | 0.43 - 2.22 | 0.9 |
